# Changes in mandibular movement during chewing of different hardness foods

**DOI:** 10.1007/s10266-016-0292-z

**Published:** 2017-02-01

**Authors:** Marie Komino, Hiroshi Shiga

**Affiliations:** Department of Partial and Complete Denture, The Nippon Dental University School of Life Dentistry, 1-9-20 Fujimi, Chiyoda-Ku, Tokyo 102-8159 Japan

**Keywords:** Masticatory movement, Masseter muscular activity, Food hardness, Gummy jelly

## Abstract

In order to clarify the change in mandibular movement during chewing of foods with different hardness, 20 healthy subjects were asked to chew 3 types of gummy jellies (containing 6, 8, and 10% gelatin), and the masseter muscular activity and the mandibular movement were recorded. The indicators representing the muscular activity (integral value of masseter muscular activity), the mandibular movement (opening distance, masticatory width, cycle time, opening maximum velocity and closing maximum velocity), and the stability of masticatory movement were calculated, respectively, and compared among the three foods. The integral value of masseter muscular activity was smallest for the 6% gelatin and significantly increased in order as the content of gelatin increased to 8, 10%. The value of each indicator for the mandibular movement increased gradually as the food got harder. The value for all indicators was significantly larger for the 10 than the 6%. However, between the two foods, no significant change was observed for the several indicators. The mean ratio of the 10% gelatin to the 8% gelatin for the cycle time was extremely small, being 1.01, but was between the range of 0.89–1.07 showing aspects of changes within each individual. The other indicators showing small ratio were similar in this aspect. The parameters representing stability of movement showed the lowest values for the 8% gelatin. It was suggested that the hardness of food affected mandibular movement during mastication, but the movement changed variously according to the hardness and exerted muscular activities.

## Introduction

Although mastication is maintained in a rhythmic pattern by the central pattern generator located in the brainstem [1], it is also autoregulated by feedback signals from peripheral sensory receptors [2–4].

It has been reported that masticatory movements are influenced by foods and vary with the type of foods, particularly with varying food textures, and many studies have been carried out to explore the effects of differences in the size and hardness of foods on the masticatory movement [5–18].

Based on the results of studies conducted to determine the effect of the size on the masticatory movement, there is unanimity of the opinion that the cycle time tends to be prolonged with increasing size of food, and the masticatory muscular activity, amount of movement, and also the velocity of movement increase [8, 9, 12, 13]. With respect to the hardness of food, there is consensus of opinion that the masticatory muscular activity increases with increasing hardness of food, although there is still lack of unanimity with respect to the influence on the cycle time, amount of movement, and velocity of movement [5–7, 10, 11, 14–18].

When viewed from the angle of test food, different types of foods were used in some of the studies carried out to determine the changes in the masticatory movement associated with different levels of hardness of foods [5–7, 16], whereas test food of the same type was used in studies conducted to determine the differences in the masticatory movements associated with different sizes [8, 9, 12, 13]. While carrying out experiments using different types of foods, which would differ in size and weight of the food, the influence of differences in the latter may have an influence on the results. Therefore, it may be desirable to carry out evaluations using a test food whose degree of hardness alone can be modified whenever necessary, and eliminate the effects of size and weight.

In this study, in order to clarify the change in mandibular movement during chewing of foods with different hardness, we analyzed the opening distance, masticatory width, cycle time, maximum velocity, and masseter muscular activity in healthy subjects while they chewed foods of varying degrees of hardness; the food was gummy jelly with same size and weight, but varying in hardness.

## Materials and methods

### Ethics statement

All the experimental procedures were approved by the Ethics Committee of Nippon Dental University (NDU–T2012–29, T2013-2015). Informed consent was obtained from all the subjects after they were received the general nature of the study.

### Subjects

Twenty healthy subjects (10 males and 10 females, 20–39 years of age, average; 26.8 years) participated in this study. None of the subjects had any clinical abnormalities in the masticatory system. The following selection criteria were applied: no complaints about bite; possession of a full complement of teeth excluding the third molars; no major dental restorations, and no history of orthodontic treatment. All the subjects were able to distinguish their habitual chewing side which was one of the requirements.

### Test food

Three types of gummy jellies having different hardness, containing 6, 8, and 10% gelatin were prepared, based on gummy jelly containing 8% gelatin which has been confirmed as being able to be masticated unconsciously [19, 20].

The test food was cylindrical in shape with a diameter of 14 mm, height of 8 mm, and weight of 2 g (Table 1).

**Table 1:** The size, weight, hardness, and ingredients of the gummy jelly

Size (mm)		ϕ 14 × 8	
Weight (g)		2	
Hardness (kPa)	69.8	109.6	150.2
Ingredient (%w/w)			
Gelatin	6	8	10
Maltose	40	40	40
Solbitol	10	10	10
Glucose	5	5	5
Others (water)	39	37	35

### Hardness of test food

**Fig. 1:**
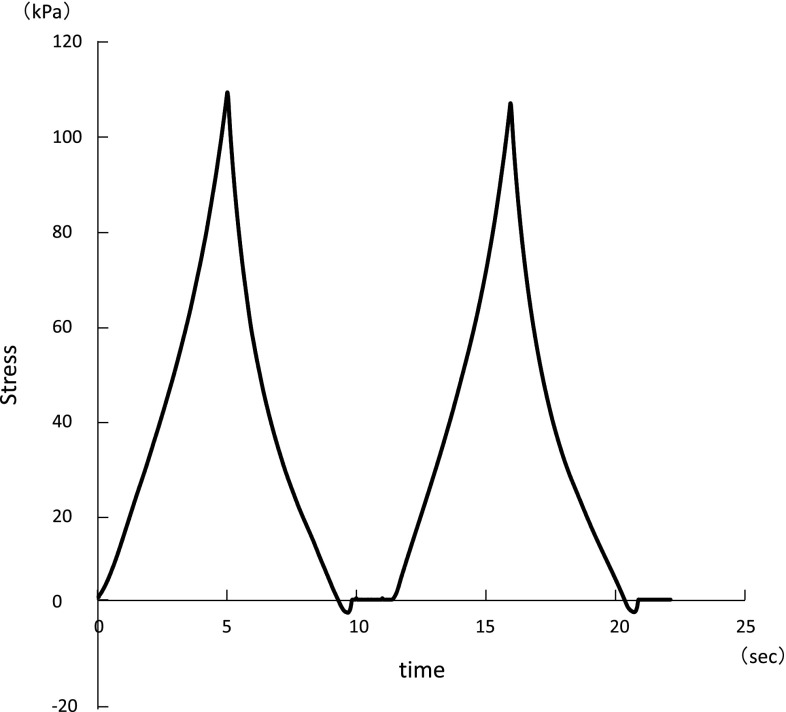
Texture profile curve for the test food. Hardness is defined as the maximum stress value of the first compression curve, (example of the gummy jelly containing 8% gelatin)

The hardness of food was measured at approximately 25 °C using a texture analyser (TA.XT PLUS, EKO, Tokyo, Japan). Compressions were performed at a constant displacement rate of 1.0 mm/s and at a compression ratio of 50% of the original sample height. Five samples for each hardness were tested and the resulting values averaged (Table 1; Fig. 1).

### Recording method

The subjects were asked to chew 3 types of gummy jellies on their habitual chewing side for 20 s. The order of the three types of food in each chewing was done randomly and 5 min interval between each mastication was set. The masseter muscular activity was recorded by surface electromyography (RM6000, Nihon Koden, Tokyo, JAPAN) for both left and right masseter muscles. The movement of the mandibular incisal point was recorded by a mandibular kinesiograph (MKG K6I, Myotronics, Seattle, WA, USA). The muscular activity and the mandibular movement were collected simultaneously using a data recorder (XR-5000, TEAC, Tokyo, JAPAN). Indicators representing the masseter muscular activity and movement of the mandibular incisal point were established as below.

### Masseter muscular activity

**Fig. 2:**
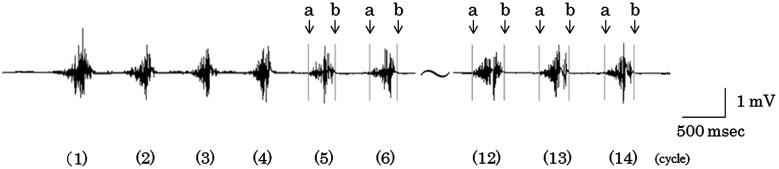
Masseter electromyograph of the 1st–6th and the 12th–14th cycles. **a** beginning of electrical discharge, **b** ending of electrical discharge, (example of the subject 1)

For the masseter muscular activity, the analog signals from the data recorder were converted into digital signals at 2,000 Hz. For the ten cycles from the fifth cycle of mastication, the integral value of muscular activities of each cycle on the habitual chewing side was calculated by establishing on the display, using a mouse, the beginning and the ending of electrical discharge of each masticatory cycle’s muscular activities (Fig. 2). Then the mean value of ten cycles was used as the indicator.

From the research [21] that we have done in the past, the results of investigating the amount of movement, cycle time, and muscular activities on the first 10 cycles of masticating various types of food showed that the changes were large for the first few cycles of mastication and thereafter the changes became smaller. Also, it has been confirmed that in order to evaluate the stability of the movement it is most suitable to use the 10 cycles from the fifth cycle of mastication [9, 22]. Then, in this study the 10 cycles from the 5th cycle were chosen.

### Movement of the mandibular incisal point

For the mandibular incisal point, the analog signals from the data recorder were converted into digital signals at 100 Hz and the indicators representing the path, rhythm, and velocity of masticatory movement, and stability of masticatory movement path and rhythm for ten cycles from the fifth cycle were calculated. The indicators were calculated as follows.

- Masticatory movement path and stability of masticatory movement path

**Fig. 3:**
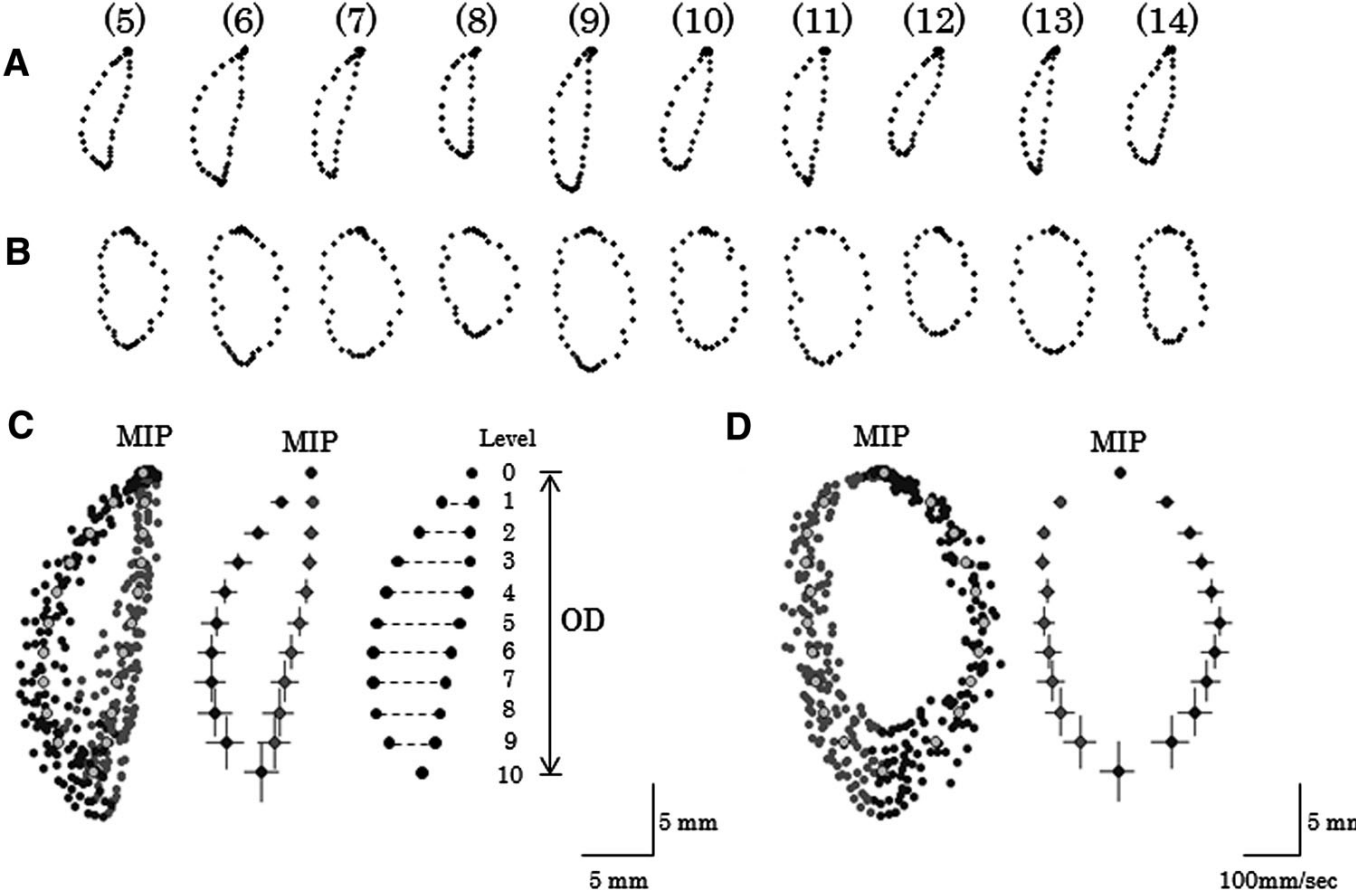
Method used to calculate the average path. **a** Ten cycles consisting of the vertical component and lateral component, **b** Ten cycles consisting of the vertical component and velocity component, **c** Overlapping of each cycle of A and average path and SDs of each level, **d** Overlapping of each cycle of B and average path and SDs of each level. *MIP* maximum intercuspal position, level 0: MIP, level 10: maximum jaw opening,—: masticatory width, (example of the subject 1)

The average path was calculated from the opening and closing paths, consisting of vertical and lateral components, of mandibular movement in the 10 cycles from the 5th cycle [9, 23] (Fig. 3). The opening distance and masticatory width were used as indicators representing the masticatory movement path. The opening distance was defined as the vertical distance from the maximum intercuspal position (MIP, level 0) to the maximum jaw opening (level 10), and the masticatory width was defined as the average width from the first to the ninth level. The average of the 11 standard deviations (SDs) from level 0 to the 10th level in the horizontal direction during opening movement, in the horizontal direction during the closing movement, and in the vertical direction were calculated as the opening lateral component, closing lateral component and vertical component, respectively. Then, these values were divided by the opening distance, and the resulting values (standard deviation/opening distance, SD/OD) were used as the three indicators representing the stability of the masticatory path (Table 2).

**Table 2:** Numerical data of the average path and velocity component

Level	Lateral component (mm)				Width (mm)	Vertical component (mm)		Velocity component (mm/sec)			
	Opening		Closing					Opening		Closing	
	Mean	SD	Mean	SD		Mean	SD	Mean	SD	Mean	SD
0	0.0	0.1	0.0	0.1		0.0	0.1	0.0	6.0	0.0	6.0
1	−0.1	0.4	2.0	0.6	2.1	1.9	0.2	124.3	13.1	94.8	23.0
2	−0.1	0.4	3.6	0.8	3.7	3.9	0.4	158.3	15.8	145.2	23.9
3	0.0	0.5	4.9	0.9	4.9	5.9	0.6	160.7^f^	14.6	167.9	24.9
4	0.3	0.5	5.9	0.9	5.6	7.9	0.8	152.0	18.5	190.3	24.5
5	0.8	0.7	6.5	0.9	5.7	9.9	0.9	156.3	26.3	204.7^g^	18.7
6	1.3	0.8	6.8	0.9	5.5	11.9	1.1	147.3	21.9	196.9	19.4
7	1.7	0.9	6.8	1.0	5.1	13.9	1.3	141.3	14.8	179.1	7.1
8	2.2	1.0	6.6	1.2	4.4	15.8	1.5	124.0	15.6	155.8	18.4
9	2.5	1.1	5.8	1.3	3.3	17.8	1.7	82.9	18.6	105.0	20.8
10	3.4	1.2	3.4	1.2		19.8^a^	1.9	4.7	9.3	4.7	9.3
Mean		0.73		0.93	4.5^b^		1.00	138.6		160.0	
SD/OD (%)		3.69^c^		4.70^d^			5.05^e^				

^a^Opening distance (OD)

^b^Masticatory width

^c^Standard deviation/opening distance (SD/OD) of opening lateral component

^d^SD/OD of closing lateral component

^e^SD/OD of vertical component

^f^Opening maximum velocity

^g^Closing maximum velocity

- Masticatory movement rhythm and stability of masticatory movement rhythm

For 10 cycles from the fifth cycle the cycle time was calculated. And then the coefficient of variation (CV) was obtained from the mean time of the ten cycles and its standard deviation, and the mean value was used as masticatory movement rhythm, and the CV was used to represent the stability of masticatory movement rhythm.

### Masticatory movement velocity

From the opening and closing paths consisting of vertical component and velocity component, the average path was calculated as well as masticatory movement path (Fig. 2). The maximum values of opening and closing movement on the average path were used as opening maximum velocity and closing maximum velocity (Table 2).

### Statistical analysis

The indicators representing the masseter muscular activity and the mandibular movement were compared among the 3 types of foods with different hardness. All the data were analyzed with statistical software (SPSS for Windows 10.0 J, SPSS, Chicago, IL, USA). The significance of differences among the 3 types of foods was evaluated by analysis of variance (ANOVA). When significant effects were identified, Bonferroni’s multiple comparison test was performed. All statistical analyses were performed with significance set at the 0.05 and 0.01 probability levels.

## Results

The integral value of masseter muscular activity was smallest for the 6% gelatin and increased in order as the content of gelatin increased to 8, 10%. A significant difference was found between all three foods (Table 3).

**Table 3:** *F* ratio (ANOVA), mean values and their standard deviations for the values of the masticatory movement path, masticatory movement rhythm, masticatory movement velocity, and muscular activity with differing gelatin concentrations

	*F* ratio	6%	8%	10%	*P* value		
					6–8%	6–10%	8–10%
Masticatory movement path							
Opening distance (mm)	67**	15.6 ± 1.6	18.1 ± 2.3	18.4 ± 2.3	0.000	0.000	0.729
Masticatory width (mm)	34**	2.7 ± 0.6	2.8 ± 0.6	3.4 ± 0.8	0.705	0.000	0.000
Masticatory movement rhythm							
Cycle time (msec)	7*	603.5 ± 61.9	620.2 ± 73.3	622.7 ± 56.6	0.059	0.002	0.646
Masticatory movement velocity							
Opening maximum velocity (mm/sec)	25**	142.5 ± 29.8	159.7 ± 33.5	168.5 ± 35.5	0.000	0.000	0.072
Closing maximum velocity (mm/sec)	28**	127.9 ± 24.6	143.3 ± 26.3	151.7 ± 25.6	0.000	0.000	0.053
Muscular activity							
Integral value of masseter muscular activity (mV msec)	70**	28.2 ± 11.3	33.2 ± 14.4	39.0 ± 15.6	0.000	0.000	0.000

* *P* < 0.05, ** *P* *<* 0.01

The value of each indicator for the masticatory movement path, the masticatory movement rhythm, and the masticatory movement velocity increased gradually as the food got harder. The value for all indicators was significantly larger for chewing food containing 10% gelatin than chewing food containing 6% gelatin (Table 3). However, between the food containing 6% gelatin and the food containing 8% gelatin, significant change was observed for the opening distance and the maximum velocity of opening and closing, but no significant change was observed for the masticatory width and the cycle time. Between food containing 8% gelatin and food containing 10% gelatin, significant change was observed for the masticatory width, but no significant change was observed for the other indicators (Table 3).

The mean ratio of the 6% gelatin to the 8% gelatin was 0.87, 0.90, 0.90 for the opening distance, the opening maximum velocity, and the closing maximum velocity, respectively, but was small for the masticatory width and the cycle time being 0.96 and 0.98, respectively. The mean ratio of the 10% gelatin to the 8% gelatin was 1.21 for the masticatory width, but was small for the opening distance, the cycle time, the opening maximum velocity, and the closing maximum velocity being 1.02, 1.01, 1.06, 1.07. The mean ratio of the 10% gelatin to the 8% gelatin was extremely small being 1.01, but was between the range of 0.89–1.07 showing aspects of changes within each individual. The other indicators showing small ratio were similar in this aspect (Table 4).

**Table 4:** Ratio of the 6% to the 8% or the 10% to the 8% and the number of the ratio which are more or less than 1

	Masticatory movement path				Masticatory movement rhythm		Masticatory movement velocity			
	Opening distance		Masticatory width		Cycle time		Opening maximum velocity		Closing maximum velocity	
	6%/8%	10%/8%	6%/8%	10%/8%	6%/8%	10%/8%	6%/8%	10%/8%	6%/8%	10%/8%
Mean	0.87	1.02	0.96	1.21	0.98	1.01	0.90	1.06	0.90	1.07
Range	074–0.96	0.95–1.10	0.81–1.14	0.85–1.46	0.90–1.07	0.89–1.07	0.74–1.06	0.85–1.32	0.68–1.10	0.90–1.27
>1	0	11	8	19	9	11	3	16	3	15
<1	20	9	12	1	11	9	17	4	17	5

The parameters representing stability of movement showed the lowest values for the 8% gelatin and increased in the order of the gelatin content of 6% and 10% (Table 5). All the parameters representing stability of movement showed the lowest values for the food with a gelatin content of 8%, with the values tending to increase in the order of the gelatin content of the food of 6% and 10%. Significant differences in these parameters were found between the 8% and the 10% in all the parameters, between the 6% and the 8% in the opening lateral component, and between the 6% and the 10% in the vertical component and the cycle time (Table 5).

**Table 5:** *F* ratio (ANOVA), mean values and their standard deviations for the values of the stability of masticatory movement path and the stability of masticatory movement rhythm

	*F* ratio	6%	8%	10%	*P* value		
					6%–8%	6%–10%	8%–10%
Stability of masticatory path							
Opening lateral (%)	9**	4.3 ± 1.6	3.4 ± 1.9	4.9 ± 1.5	0.012	0.191	0.000
Closing lateral (%)	4*	4.5 ± 1.9	3.7 ± 1.4	5.1 ± 1.6	0.172	0.121	0.001
Vertical (%)	9**	5.1 ± 1.4	4.6 ± 1.6	6.3 ± 2.1	0.146	0.002	0.000
Stability of masticatory movement rhythm							
Cycle time (%)	13**	5.3 ± 1.9	4.6 ± 1.7	6.4 ± 2.4	0.133	0.005	0.000

* *P* < 0.05, ** *P* *<* 0.01

## Discussion

It has been reported that the muscular activity increases progressively with increasing hardness of foods [11, 14–18]. The result of this study also showed that the masseter muscular activity increased progressively with increasing hardness of the food, with significant differences between each pair of foods examined, consistent with previous reports [11, 14–18]. This may be interpreted as indicating that the chewing force increases in strength with increasing hardness of a food.

Many reports have demonstrated that the amount of masticatory movement increases with increasing hardness of the food [6, 7, 10, 11, 15–18]. In this study, the amount of movement was found to be the smallest for the food with the least gelatin content of 6% and progressively increased with increasing the food gelatin content (8 and 10%). However, for the opening distance between the 8 and the 10%, and for the masticatory width between the 6 and the 8%, no significant differences were found between the two. This indicated that according to the hardness of food, there were differences in changes in the amount of vertical and lateral masticatory movements. It is easy to see the differences by the ratio. The ratio of the 6% to the 8% for the opening distance was 0.87 and a bit smaller than the ratio (0.96) for the masticatory width. On the other hand, the ratio of the 10% to the 8% for the masticatory width was 1.21 and a bit larger than the ratio (1.02) for the opening distance. On the other hand, the ratio of 10 to 8% was 1.02 for the opening distance and a bit larger, 1.21 for the masticatory width. This could be thought that the vertical masticatory movement increased when the hardness of food was relatively soft, and the lateral masticatory movement increased when the food got harder.

Kitashima et al. [24] investigated mandibular movements during the process of mastication of hard gummy jelly in terms of the movement stages, that is, the initial, middle, and final stages, and reported that both the opening distance and the masticatory width were the greatest in the initial stage, decreasing in the middle to the final stage. They also reported that while there was no significant difference in the opening distance between the initial and middle stages of mastication, the masticatory width differed significantly between these two stages. These results indicate that it is the amount of lateral movement rather than that of vertical movement which increases during mastication of hard foods. In this study, we obtained similar results from a comparison of gummy jelly with gelatin contents of 8 and 10%.

Unlike other researchers, Pröschel et al. [5]. reported that the opening distance decreased whereas the masticatory width increased during mastication of hard foods. However, this may be explained as follows: because Pröschel et al. [5] used very hard wine gums as their hard food, the subjects needed to exert a stronger force of mastication via increase of the amount of lateral movement rather than that of vertical movement. These findings led us to conclude that while both the vertical and lateral movements increase with increasing hardness level of a test food (within the range of hardness that can be chewed normally), the degree of increase differs between the vertical and lateral movements depending on the hardness level of the food and that changes in the degrees of vertical and lateral movements vary among individuals.

There is no consensus as to cycle time, in that some reports suggest prolongation of the cycle time with increasing hardness of food [5, 6, 11, 15], while others have documented no appreciable change in the cycle time even for foods of higher hardness levels [10, 14, 16]. On closer scrutiny, however, all the results of the previous studies show a tendency for the cycle time to be longer during the chewing of hard food than during that of soft food. Kitashima et al. [24] examined the cycle time for mastication of hard gummy jelly and reported that there was no significant difference in the cycle time among the stages of mastication, but that the cycle time in the initial stage, when the food was still hard, tended to be longer than that in the middle and final stages. Slavicek et al. [25] investigated the movement for mastication of three types gummy jellies created by changing the amount of gelatin and reported that the number of chewing strokes varied only marginally.

The result of this study revealed that the cycle time was prolonged progressively with increasing hardness of the food, with a significant difference between the 6 and the 10%. Based on these findings, it is presumed that the cycle time usually increases as the hardness level of the food increases. However, unlike other indices in this study, a significant difference in the cycle time was not found between the 6 and the 8% or between the 8 and the 10%. The mean ratio of cycle time for gummy jellies with gelatin contents of 6 and 8% and for those with gelatin contents of 8 and 10% were approximately 1.0. This may indicate that the amount of change in the cycle time to maintain this masticatory rhythm is low, because the basic masticatory rhythm is set by the pattern generator in the brain stem.

However, the ratio ranged from 0.90 to 1.07 for gummy jellies with gelatin contents of 6 and 8% and 0.89–1.07 for those with gelatin contents of 8 and 10%. This shows that the cycle time was approximately 1.0, on average, because there were subjects in whom the cycle time increased and those in whom the cycle time decreased, and not because the cycle time remained unchanged irrespective of differences in the hardness level of the food. Thus, it is presumed that there are considerable changes within a particular subject depending on the hardness level of the food. However, based on the finding that the cycle time for chewing gummy jelly with a gelatin content of 10% was significantly longer than that for chewing gummy jelly with a gelatin content of 6%, it seems that the cycle time varies according to differences in the hardness level of the food and basically increases as the hardness level increases.

It has been reported by various researchers, other than Pröschel et al. [5], that the movement velocity increases with increasing hardness level of the food. In this study also, the movement velocity increased with increase in the hardness level of the food, and there was a significant difference between the movement velocities for gummy jellies with gelatin contents of 6 and 8%, and for those with gelatin contents of 6 and 10%.

These results were consistent with those for changes in the opening distance. However, when the ratio was investigated, the ratio of the 10 to the 8% was less than 1 in about a half of the subjects (9 of 20 subjects). In contrast, the ratio of the maximum opening velocity was less than 1 in only 4 subjects and that of the maximum closing velocity was less than 1 in only 5 of the 20 subjects, showing a pattern slightly different from that for the opening distance. Thus, although there was no significant difference, it became apparent that the velocities increased in the majority of subjects. It was found that the amount of lateral movement, represented by the masticatory width, increased with increase of the gelatin content of the gummy jelly from 8 to 10%. These findings suggest that the muscular activity increases with increasing hardness level of the food via augmentation of the amount of movement and the movement velocity, to exert a stronger force of mastication, although there were individual differences.

There is no report yet, to the best of our knowledge, on the stability of the masticatory movement in relation to hardness level of foods so that no comparison can be made in this respect. In this study, all the parameters reflecting stability of movement showed the lowest values for the food with a gelatin content of 8%, with the values tending to increase in the order of the gelatin content of the food of 6 and 10%. These findings are considered to indicate the existence of an optimal degree of hardness of foods.

## Conclusion

To clarify the changes in the mandibular movements during mastication in relation to varying hardness levels of the test food, we analyzed the opening distance, masticatory width, cycle time, movement velocity, and masseter muscle activity in healthy subjects during mastication of gummy jellies of varying hardness levels. The results suggest that in response to differences in the hardness level of the food, masseter muscular activity sufficient for the hardness of the test food is exerted through alteration of the pathway, rhythm, and velocity of the masticatory movements.
